# A retrospective analysis of the effect of scraping therapy with Astragalus membranaceus ointment combined with Tuina on upper limb spastic hemiplegia after hemorrhagic stroke

**DOI:** 10.12669/pjms.42.1.13842

**Published:** 2026-01

**Authors:** Xiuli Shi, Lingna Bei, Chun Chen

**Affiliations:** 1 Xiuli Shi Department of Neurosurgery, Suzhou Hospital of Integrated Traditional Chinese and Western Medicine, Suzhou, Jiangsu Province 215000, P.R. China; 2 Lingna Bei Department of Neurology, Suzhou Hospital of Integrated Traditional Chinese and Western Medicine, Suzhou, Jiangsu Province 215000, P.R. China; 3 Chun Chen Department of Orthopedics, Suzhou Hospital of Integrated Traditional Chinese and Western Medicine, Suzhou, Jiangsu Province 215000, P.R. China

**Keywords:** Hemiplegia, Hemorrhagic stroke, Huang Qi ointment, Massage, Scraping therapy, Spastic, Upper limb

## Abstract

**Objective:**

Exploring the clinical efficacy of scraping therapy with Astragalus membranaceus (AMS, Huang Qi) ointment combined with tuina (Chinese Therapeutic Massage) in patients with upper limb spastic hemiplegia after hemorrhagic stroke.

**Methodology:**

This retrospective analysis examined clinical data of 106 patients with upper limb spastic hemiplegia after hemorrhagic stroke who received treatment at Suzhou Integrated Traditional Chinese and Western Medicine Hospital from January 2023 to June 2025. According to different treatment methods, patients were divided into a tuina group (n = 53, tuina massage treatment) and a scraping + tuina group (n = 53, scraping therapy with AMS ointment combined with tuina). The modified Ashworth Spasm Assessment Scale (MAS) was used to evaluate the degree of spasticity in the affected limb. The Simplified Fugl Meyer Upper Limb Function Assessment Scale (FMA-UE) was used to assess the motor function of the affected limb and the Modified Barthel Index Assessment Scale (MBI) was used to evaluate Activities of Daily Living (ADL).

**Results:**

After treatment, the improvement in the MAS score in the scraping + tuina group was better than that in the tuina group (*P*<0.05). The post-treatment FMA-UE score and MBI score of both groups of patients significantly improved and were markedly higher in the scraping + tuina group compared to the tuina group (*P*<0.05). There was no statistically significant difference in the incidence of adverse reactions between the two groups (*P*>0.05).

**Conclusions:**

Scraping therapy with AMS ointment combined with tuina massage treatment can effectively alleviate upper limb spasticity. The combined regimen is associated with improved motor function and ADL in patients with upper limb spastic hemiplegia after hemorrhagic stroke.

## INTRODUCTION

Despite improvements in public health policies and healthcare services, the burden of stroke in China remains high.^1^ Hemorrhagic stroke is one of the main types of stroke and is closely related to factors such as hypertension, cerebral vascular malformation and aneurysms.^1^,^2^ According to statistics, in 2019 alone, 43600 out of 28.76 million stroke patients had cerebral hemorrhage.^2^,^3^ Spasmodic hemiplegia is the most common complication among stroke survivors, with a three-month incidence of about 20% and a one-year incidence rate of about 44%.^4^,^5^ In 1980, Lance first defined spasticity as one of the manifestations of the upper motor neuron syndrome, which is a velocity-dependent exaggeration of the stretch reflex.^6^,^7^ Spasticity more frequently impacts the upper limbs, often leading to the loss of upper limb motor function and severely affecting the ability of patients to perform daily activities.^6^-^8^

Currently, there are numerous clinical treatment regimens for post-stroke spasticity, including pharmacotherapy, physical therapy, rehabilitation training and surgical treatment.^8^-^11^ Pharmacotherapy primarily consists of oral medications (such as baclofen and tizanidine) and local injection medications (such as botulinum toxin).^9^ Physical therapy, which offers advantages such as safety and cost-effectiveness, is the most commonly used adjuvant treatment.^10^ It mainly includes neuromuscular electrical stimulation, vibration therapy and thermal stimulation therapy.^10^ Rehabilitation training encompasses proper limb positioning, stretching therapy and exercise therapy.^11^ Surgical treatment is rarely used in clinical practice due to its high operational difficulty, significant trauma, long post-operative recovery period and the risk of complications such as infection and joint instability.^9^-^11^

According to traditional Chinese medicine (TCM), spastic paralysis after stroke is caused by a deficiency of qi and blood, blood stasis blocking the collaterals and phlegm turbidity stagnation.^12^ Tuina (Chinese Therapeutic Massage) therapy has a long history of application in the rehabilitation of hemiplegia after stroke. By applying manual techniques to the body’s surface acupoints and meridians, it regulates the circulation of qi and blood and improves local blood circulation.^12^,^13^ However, Tuina therapy is insufficient in relieving intractable spasm. Scraping therapy is based on the TCM theory of “dredging meridians and collaterals, promoting blood circulation to remove blood stasis”. By scraping along the meridians on the body surface with a massage tool, it causes “sha” (a reddish or purplish skin rash) to appear, promoting local qi and blood circulation and improving microcirculation.^14^ Astragalus membranaceus (AMS, Huang Qi) ointment, a TCM preparation with AMS (Huang Qi) as the main raw material, was shown to be effective in replenishing qi and lifting yang, consolidating the superficial resistance to stop sweating, promoting diuresis to reduce edema and supporting toxin discharge to encourage tissue regeneration.^15^

From a biomedical perspective, scraping therapy can be understood as instrument-assisted soft tissue mobilization (IASTM), a modality that delivers controlled mechanical stimulation to modulate fascial tone, improve microcirculation, and reduce neuromuscular hyperactivity.^16^ Similarly, tuina therapy is comparable to manual myofascial stimulation and soft tissue manipulation, which facilitates proprioceptive input, reduces muscle spindle excitability, and enhances reflex neuromodulation.^8^ These effects may help improve upper-limb motor control and alleviate post-stroke spasticity through peripheral and spinal regulatory mechanisms.^17^

At present, there are no reports on the use of scraping therapy with AMS ointment combined with tuina massage for upper limb spastic hemiplegia after hemorrhagic stroke. Therefore, this study aimed to fill this gap and to clarify the clinical efficacy of scraping therapy with AMS ointment combined with tuina massage for patients with upper limb spastic hemiplegia after hemorrhagic stroke.

## METHODOLOGY

This retrospective analysis was conducted at Suzhou Hospital of Integrated Traditional Chinese and Western Medicine. A total of 106 patients with upper limb spastic hemiplegia after hemorrhagic stroke, who received treatment from January 2023 to June 2025, were enrolled. These patients were consecutively selected from the hospital’s electronic medical record system, minimizing the risk of selection bias. Among them, 53 patients received Tuina therapy (the tuina group) and the 53 patients received scraping therapy with AMS ointment combined with tuina (the scraping + tuina group).

### Ethical approval:

This retrospective study was approved by the Ethics Committee of Suzhou Hospital of Integrated Traditional Chinese and Western Medicine (Approval No. 2025-037; Date: September 13th, 2025). Informed consent was waived in accordance with institutional regulations, as the study used de-identified data extracted from existing medical records and posed minimal risk to participants. All patient information was anonymized prior to analysis to ensure confidentiality.

### Inclusion Criteria:

- Meeting the diagnostic criteria for hemorrhagic stroke as specified in the Chinese Guidelines for the Management of Intracerebral Hemorrhage (2021) and confirmed by cranial computed tomography (CT) or magnetic resonance imaging (MRI).
- First onset of the disease with a course of ≤ six months.
- A Modified Ashworth Scale (MAS) score of ≥ 1 in the affected upper limb prior to intervention, as assessed by qualified rehabilitation physicians.
- Age ≤ 75 years.

### Exclusion Criteria:

- Complicated with severe dysfunction of the heart, liver, kidney or other vital organs.
- Upper limb skin damage, infection or bleeding tendency (e.g., abnormal coagulation function).
- Complicated with other neurological diseases.
- A history of underlying diseases such as upper limb fracture and arthritis.

### Intervention Protocol:

All patients received routine medication and rehabilitation nursing care. The patients underwent rehabilitation training conducted by professional rehabilitation therapists. The training included active/passive flexion, extension, internal and external rotation of the affected side joints. Each treatment session lasted 15 minutes, once a day, five times a week, for a consecutive 45 days.

Relaxation Manipulations: The patient took a supine position. The therapist applied rolling and kneading manipulations to the affected upper limb (from the shoulder to the hand), focusing on relaxing the biceps brachii, flexor carpi muscles and flexor digitorum muscles. Each part was manipulated for five minutes until the muscles were relaxed.

The therapist pressed the affected side acupoints, including Quchi (the depression at the lateral end of the cubital crease), Shousanli (2 cun below Quchi), Hegu (the midpoint on the radial side of the 2^nd^ metacarpal bone on the dorsum of the hand) and Neiguan (2 cun above the wrist crease, between the palmaris longus tendon and the flexor carpi radialis tendon). Each acupoint was pressed for 30 seconds, with the intensity appropriate for the patient to feel soreness and distension.

Based on relaxation, the therapist slowly moved the affected shoulder joint (forward flexion, abduction, internal rotation), elbow joint (flexion and extension) and wrist joint (dorsiflexion and palmar flexion). Each joint was moved five times and excessive force that might cause pain was avoided.

The therapist applied rubbing manipulations to the affected upper limb, moving back and forth from the shoulder to the hand for three minutes until the skin felt warm. Tuina therapy was performed by therapists with more than five years of experience in tuina rehabilitation. Each session lasted 30 minutes, once a day, five times a week, for a consecutive 45 days.

### Scraping therapy with AMS ointment:

The ointment was prepared and supplied by the Pharmacy Department of Suzhou Hospital of Integrated Traditional Chinese and Western Medicine, following a standardized formulation protocol. Each batch was produced using fixed proportions of certified raw herbs: 30g of Astragalus membranaceus (Huang Qi), 15g of Angelica sinensis (Danggui), 15g of Carthamus tinctorius (Honghua), 15g of Boswellia carterii (Ruxiang), and 15g of Commiphora myrrha (Moyao), incorporated into 100g of petrolatum. All herbal components were sourced from GMP-certified suppliers and conformed to the Chinese Pharmacopoeia (2020 edition). Although quantification of active compounds (e.g., via HPLC) was not performed due to the retrospective nature of the study, batch-to-batch consistency was ensured through the use of standardized granules and uniform weighing and processing procedures. The mixture was heated, stirred evenly, and cooled at room temperature to yield a homogeneous ointment.

The selected meridians included the Large Intestine Meridian of Hand-Yangming, the Triple Energizer Meridian of Hand-Shaoyang and the Lung Meridian of Hand-Taiyin. The specific parts were as follows: Large Intestine Meridian of Hand-Yangming, from Jianyu acupoint (on the shoulder) to Hegu acupoint (on the hand); Triple Energizer Meridian of Hand-Shaoyang, from Jianliao acupoint (on the shoulder) to Zhongzhu acupoint (on the hand); Lung Meridian of Hand-Taiyin, from Chize acupoint (on the elbow) to Taiyuan acupoint (on the hand).

The patient was guided into a sitting position, with the affected upper limb exposed. AMS ointment was evenly applied to the scraping area (with a thickness of approximately 1mm). A horn scraper (with a smooth edge) was used to scrape along the direction of the meridians at a 45° angle. The intensity was increased from light to heavy (appropriate for the patient’s tolerance without causing pain) and the scraping speed was about 3cm/s. Each area was scraped until light red “sha” (a reddish skin rash) appeared on the skin (dark ecchymosis was avoided). Each meridian was scraped for five minutes.

After scraping, the residual ointment was wiped off with a clean towel. The patients were advised to avoid contact with cold water for one hour, keep warm and prevent cold exposure. Each scraping session lasted 20 minutes and was conducted simultaneously with tuina therapy (tuina was first performed for relaxation, followed by scraping and finally tuina again for consolidation). The treatment was administered once a day, four times a week, for a consecutive 45-day period. The treatment frequency in the scraping + tuina group was limited to four times per week due to institutional clinical guidelines, which recommend restricting scraping sessions to no more than four times weekly to allow for adequate skin recovery. Tuina was applied concurrently with scraping during each session. According to the medical records, all patients completed the full 45-day course of treatment without dropout. No cases of treatment refusal or discontinuation due to discomfort, skin rash, or other adverse events were observed. While no formal satisfaction survey was conducted due to the retrospective nature of the study, the mild and transient adverse effects recorded suggest high patient adherence and good tolerability of the intervention.

### Outcome measures:

The Modified Ashworth Scale (MAS) was used to evaluate the spasm severity of the affected upper limb. The grading criteria were as follows: Grade 0: No increase in muscle tone; Grade 1: Slight increase in muscle tone, with mild resistance appearing in the last 1/4 of the range of motion (ROM); Grade 1+: Slight increase in muscle tone, with mild resistance appearing in the last 1/2 of the ROM; Grade 2: Significant increase in muscle tone, with resistance appearing in most parts of the ROM, but the joint can still move; Grade 3: Severe increase in muscle tone, making joint movement difficult; Grade 4: Extreme increase in muscle tone, with joint rigidity. The MAS grades of the two groups were recorded before treatment and after 45 days of treatment and a decrease in grade was regarded as spasm relief.

The Fugl-Meyer Assessment of Upper Extremity (FMA-UE) was adopted to evaluate the motor function of the affected upper limb. This scale covers 10 dimensions, including reflex activity, flexor synergy movement, extensor synergy movement, wrist joint movement and finger movement, with a total of 33 items. Each item is scored as “0 points (unable to complete), 1 point (partially completed), 2 points (fully completed)” and the total score ranges from 0 to 66 points. A higher score indicates better upper limb motor function.

The Modified Barthel Index (MBI) was used to assess the ADL. MBI includes 10 items such as eating, washing, dressing, using the toilet and walking. The total score ranges from 0 to 100 points, with the following classifications: 0-20 points: extremely severe functional impairment; 21-40 points: severe functional impairment; 41-60 points: moderate functional impairment; 61-99 points: mild functional impairment; 100 points: normal function. A higher score indicates stronger ADL ability.

### Statistical analysis:

All statistical analyses were conducted using SPSS version 26.0 (IBM Corp., Armonk, NY, USA). The Shapiro–Wilk test was applied to assess the normality of continuous variables. Normally distributed data were presented as mean ± standard deviation (SD), while non-normally distributed data were expressed as median and interquartile range (IQR). For between-group comparisons, independent samples t-tests were used for normally distributed variables, and the Mann–Whitney U test was used for non-parametric and ordinal data, including MAS scores. For within-group comparisons, paired t-tests were used for normally distributed data, and the Wilcoxon signed-rank test was applied for non-parametric and ordinal variables. Categorical data were summarized as frequencies and compared using the chi-square test. A two-tailed P-value < 0.05 was considered statistically significant.

## RESULTS

This study included 106 patients (67 males and 49 females) with an age range of 40-75 years and an average age of 61.1 ± 8.5 years. There was no statistically significant difference in the comparison of baseline data between the groups (*P*>0.05) (Table-I).

**Table-I:** Comparison of baseline data between two groups of patients.

Baseline data	Scraping + tuina (n=53)	Tuina (n=53)	χ²² / t/ Z	P
Male, n(%)	31 (58.5)	36 (67.9)	1.01	0.314
Age (years), mean±SD	62.1±8.6	60.2±8.3	1.16	0.269
BMI (kg/m²), mean±SD	23.9±2.9	23.2±3.0	1.22	0.239
Hypertension, n(%)	21 (39.6)	25 (47.2)	0.61	0.433
Diabetes, n(%)	26 (49.1)	30 (56.6)	0.61	0.436
Course of disease (days), M(IQR)	69 (34-98)	58 (34-74)	1.36	0.173
Affected side, n(%)			0.34	0.56
Left	27 (50.9)	26 (49.1)		
Right	26 (49.1)	27 (50.9)		

Before treatment, there was no statistically significant difference in MAS ratings between the two groups of patients (P>0.05). After 45 days of treatment, both groups showed better improvement in MAS ratings than before treatment and the scraping + tuina group had a better MAS rating than the tuina group (P<0.05) (Table-II). Specifically, the MAS grades in both groups decreased significantly after treatment (P<0.001 in both groups, Wilcoxon signed-rank test), indicating reduced spasticity. The median MAS score in the scraping + tuina group improved from 2 (IQR: 2–2) to 1 (IQR: 1–2), while in the tuina group it changed from 2 (IQR: 1–2) to 1 (IQR: 1–2).

**Table-II:** Comparison of MAS ratings between two groups of patients.

Time	MAS	Scraping + tuina (n=53)	Tuina (n=53)	χ²	P
Before treatment	Grade-0	0 (0)	0 (0)	1.16	0.884
	Grade-1	1 (1.9)	0 (0)		
	Grade-1+	12 (22.6)	11 (20.8)		
	Grade-2	24 (45.3)	24 (45.3)		
	Grade-3	9 (17.0)	10 (18.9)		
	Grade-4	7 (13.2)	8 (15.1)		
After 45 days of treatment	Grade-0	13 (24.5)	4 (7.5)	12.02	0.035
	Grade-1	20 (37.7)	13 (24.5)		
	Grade-1+	9 (17.0)	19 (35.8)		
	Grade-2	9 (17.0)	11 (20.8)		
	Grade-3	1 (1.9)	3 (5.7)		
	Grade-4	1 (1.9)	3 (5.7)		

Before treatment, FMA-UE and MBI scores of the two groups were similar (*P*>0.05). After 45 days of treatment, both groups showed an increase in FMA-UE and MBI scores compared to before treatment and the scores were markedly higher in the scraping + tuina group compared to the tuina group (*P*<0.05) (Table-III).

**Table-III:** Comparison of FMA-UE and MBI scores between two groups of patients.

Time	Variables	Scraping + tuina (n=53)	Tuina (n=53)	t	P
Before treatment	FMA-UE	32.5±5.0	31.3±4.2	1.34	0.161
	MBI	47.7±6.3	49.2±7.3	-1.13	0.275
After 45 days of treatment	FMA-UE	45.5±6.6	41.3±6.0	3.43	0.001
	MBI	75.8±7.8	67.9±6.7	5.59	<0.001

As shown in Table-IV, there were seven cases of adverse reactions in the scraping + tuina group and five cases in the tuina group during the treatment process, with no statistically significant intergroup difference in the overall incidence rate (*P*>0.05).

**Table-IV:** Comparison of adverse reactions between two groups.

Adverse	Scraping + tuina (n=53)	Tuina (n=53)	χ^2^	P
Local skin redness	1 (1.89)	0 (0)	-	-
Itching and rash on the skin	2 (3.77)	1 (1.89)	-	-
Muscle soreness worsens	2 (3.77)	1 (1.89)	-	-
Dry and flaky skin	1 (1.89)	1 (1.89)	-	-
Joint pain or discomfort	1 (1.89)	2 (3.77)	-	-
Total number of occurrences	7 (13.21)	5 (9.44)	0.38	0.540

## DISCUSSION

This study demonstrated that scraping therapy with AMS ointment combined with tuina massage treatment is safe and can effectively alleviate upper limb spasticity and improve motor function and ADL in patients with upper limb spastic hemiplegia after hemorrhagic stroke.

The results of our previous study showed the efficiency of scraping therapy with AMS ointment in treating patients with upper limb spastic hemiplegia after hemorrhagic stroke.^15^ For example, in a recent prospective study, Li et al.^18^ found that combined therapy—including acupuncture, traditional Chinese herb hot compress, and rehabilitation training—led to greater improvements in shoulder–hand syndrome symptoms, upper-limb function, and activities of daily living compared with rehabilitation alone. In this study, we observed the efficacy of scraping therapy with AMS ointment combined with tuina in treating patients with upper limb spastic hemiplegia after hemorrhagic stroke. The results of this study showed that after 45 days of treatment, the MAS grade of the scraping + tuina group was significantly better than that of the tuina group. Similarly, the combined treatment led to markedly better scores of the FMA-UE and MBI. These results suggest that scraping therapy with AMS ointment combined with tuina can significantly improve the rehabilitation effect and enhance the ADL ability in patients with upper limb spastic hemiplegia after hemorrhagic stroke, without an increase in adverse reactions.

Previous studies have confirmed the efficacy of tuina therapy in the rehabilitation of post-stroke hemiplegia. Yang et al.^19^ conducted a study on 100 patients with upper limb spasticity after stroke and showed that compared with conventional rehabilitation training, tuina therapy led to more significant improvements in upper limb spasticity, limb motor function and ADL. Similarly, Liu et al.^20^ administered tuina therapy based on graded motor imagery (GMI) and demonstrated that the combination of tuina and GMI could better improve the upper limb motor function of patients with post-stroke hemiplegia and enhance their quality of life. In addition, Ma et al.^21^ adopted acupuncture combined with meridian-tendon tuina to treat patients with upper limb spastic hemiplegia after ischemic stroke.

The results indicated that this combined therapy improved upper limb spasticity in patients with ischemic stroke, while reducing muscle tone and enhancing daily motor function. However, acupuncture carries the risks of local tissue damage and infection, requires high levels of experience from operators and may cause symptoms such as tension and dizziness in some patients, leading to low compliance.^22^ In contrast, scraping therapy is characterized by simplicity of operation, safety, minimal invasiveness and high compliance.^15^,^18^ The results of this study have clearly demonstrated that incorporating scraping therapy with AMS ointment into the tuina massage treatment can bring additional benefits. This improved efficiency may be explained by several possible mechanisms. First, scraping therapy can dilate local skin capillaries through mechanical stimulation, increase blood flow, improve blood supply to the muscle tissue of the affected upper limb, enhance the delivery of oxygen and nutrients, reduce the accumulation of metabolic products such as lactic acid and thereby relieve muscle fatigue and spasticity.^14^,^23^

A meta-analysis of 23 studies by Guo et al.^23^ found that scraping therapy improved the treatment effectiveness and quality of life of patients with post-stroke hemiplegia, highlighting its positive significance for hemiplegia rehabilitation. Second, the ointment that was used as the medium for scraping contains TCM ingredients such as AMS (Huang Qi), Angelica sinensis (Danggui) and Carthamus tinctorius (Honghua). AMS is regarded as the “primary herb for replenishing qi” in TCM, as it can tonify the qi of the spleen and lung and promote the circulation of qi and blood.^24^ Angelica sinensis and Carthamus tinctorius can activate blood circulation and resolve blood stasis, assisting AMS in promoting blood flow.^25^,^26^

The microchannels formed on the skin by scraping can promote the local penetration of active ingredients of the AMS ointment, reduce the excitability of γ-motor neurons and thus alleviate the hyperactivity of the muscle stretch reflex.^23^-^26^ Meanwhile, tuina massage acts on acupoints (e.g., Quchi, Hegu) through manual manipulations to stimulate the circulation of qi and blood along meridians. It exerts a synergistic effect with scraping therapy to achieve the effects of “dredging meridians to relieve pain and reducing spasticity”.^18^,^20^,^21^

From a biomedical perspective, the combination of AMS ointment, scraping therapy, and tuina may exert therapeutic synergy through several complementary mechanisms. First, scraping induces transient microchannels in the skin, enhancing the transdermal delivery of AMS ointment. Its active herbal components—Astragalus membranaceus, Angelica sinensis, and Carthamus tinctorius—have demonstrated anti-inflammatory and microcirculation-promoting effects, which may help relieve local stiffness.^24^,^25^ Second, both scraping and tuina deliver rhythmic mechanical stimulation to the skin and underlying fascia, promoting local blood flow, reducing myofascial adhesions, and accelerating the clearance of metabolic byproducts such as lactic acid.^16^,^17^ Third, these mechanical stimuli activate cutaneous and proprioceptive receptors, modulating spinal reflex excitability via peripheral neuromodulation pathways. This can reduce γ-motor neuron hyperexcitability, which underlies spasticity.^6^-^8^ Collectively, this multimodal approach may improve neuromuscular function by combining transdermal pharmacologic action, mechanical input, and reflex circuit modulation.

Furthermore, the combined therapy demonstrates favorable feasibility for promotion in primary healthcare settings. Tuina and scraping are non-invasive, low-cost interventions that do not require complex or expensive equipment, making them accessible in resource-limited environments.^13^,^23^ In China, many community hospitals and rehabilitation institutions already offer basic training in traditional Chinese medicine therapies, including tuina and scraping, which supports their local implementation.^13^ The AMS ointment can be prepared by hospital pharmacies using standardized formulas and widely available herbal ingredients.^15^,^24^ Additionally, the required tools, such as horn scrapers, are inexpensive and reusable.^23^ Although the techniques themselves are relatively easy to master, adequate therapist training and brief patient education—particularly regarding post-scraping care and cold avoidance—remain essential to ensure safety and treatment compliance. These factors collectively support the practical application of the intervention in grassroots medical institutions.

Future research should aim to conduct large-scale, multi-center randomized controlled trials (RCTs) to validate the therapeutic efficacy of scraping therapy with AMS ointment combined with tuina. Additionally, incorporating long-term follow-up assessments is essential to determine the durability of treatment benefits. Objective physiological indicators—such as inflammatory cytokines or neurotrophic biomarkers—may help elucidate underlying mechanisms. Furthermore, neuroimaging techniques like functional MRI or diffusion tensor imaging (DTI) can be employed to explore changes in brain plasticity and neuromuscular modulation associated with the therapy. These directions may enhance the mechanistic understanding and clinical translation of this traditional intervention.

### Strength of the study:

To our knowledge, this is the first report confirming the efficacy of scraping therapy with AMS ointment combined with tuina in the treatment of patients with upper limb spastic hemiplegia due to hemorrhagic stroke.

### Limitations

First, it was a single-center retrospective analysis with a relatively small sample size, which limits the generalizability of the findings and weakens the ability to establish causal relationships. As the data were derived from existing medical records, some potentially important variables—such as lesion location, hematoma volume, and the specific timing or intensity of rehabilitation—were not consistently documented and thus could not be included in the analysis. Second, the study lacked long-term follow-up, preventing evaluation of the durability of treatment effects. Third, no standardized patient-reported outcome measures (PROMs) were collected due to the retrospective design. Although all participants completed the intervention without withdrawal, structured assessments of patient satisfaction and acceptability should be incorporated in future prospective studies to better reflect real-world experience. Fourth, laboratory and physiological indicators (e.g., inflammatory cytokines, neurotrophic factors) were not assessed, and neuroimaging tools (such as functional MRI or diffusion tensor imaging) were not utilized, limiting insights into the peripheral and central mechanisms of action. Fifth, the difference in weekly treatment frequency between groups may introduce a confounding factor. Although the combined group received fewer sessions per week, their clinical outcomes were superior, suggesting a potential synergistic effect. However, future studies should match treatment frequency across groups to enhance comparability. Despite these limitations, efforts were made to minimize bias through strict inclusion criteria (e.g., disease duration ≤6 months) and by ensuring baseline comparability in demographic and clinical variables (e.g., age, sex, MAS, FMA, MBI scores). Nevertheless, to improve scientific rigor, future research should adopt randomized controlled designs with larger multicenter cohorts, prospective data collection, inclusion of objective biomarkers, and advanced neuroimaging assessments to comprehensively evaluate therapeutic efficacy and underlying mechanisms.

## CONCLUSION

The present retrospective analysis suggests that scraping therapy combined with AMS (Huang Qi) ointment and tuina massage may be beneficial in reducing upper limb spasticity and may contribute to improvements in motor function and activities of daily living in patients with upper limb spastic hemiplegia after hemorrhagic stroke. The combined intervention was well tolerated, with no increase in adverse events observed in this cohort. Owing to its relatively simple operational requirements and low cost, the regimen may have potential for application in primary healthcare settings; however, these findings should be interpreted cautiously given the study’s retrospective design and lack of long-term follow-up.

### Funding

Guiding Project of Suzhou Science and Technology Development Plan (Basic Research Medical Application Basic Research) (SKYD2023211)

### Authors’ contributions:

**XS:** Literature search, study design and manuscript writing.

**XS, LB and CC:** Data collection, data analysis and interpretation. Critical Review.

**XS:** was involved in the manuscript revision and validation and is responsible for the integrity of the study.

All authors have read and approved the final manuscript.
